# A polycrystalline diamond micro-detector for X-ray absorption fine-structure measurements

**DOI:** 10.1107/S1600577521013011

**Published:** 2022-02-16

**Authors:** Lei Yao, Yunpeng Liu, Bingjie Wang, Lixiong Qian, Xueqing Xing, Guang Mo, Zhongjun Chen, Zhonghua Wu

**Affiliations:** a Institute of High Energy Physics, Chinese Academy of Sciences, Beijing 100049, People’s Republic of China; b College of Physics and Electronic Engineering, Mudanjiang Normal University, Mudanjiang 157000, People’s Republic of China; c University of Chinese Academy of Sciences, Chinese Academy of Sciences, Beijing 100049, People’s Republic of China

**Keywords:** polycrystalline diamond, X-ray absorption spectroscopy, X-ray detector

## Abstract

The microminiaturization of detectors used to record the intensity of X-ray beams is very favorable for combined X-ray experimental techniques. In this paper, chemical-vapor-deposited (CVD) polycrystalline diamond film was used to fabricate a micro-detector owing to its well controlled size, good thermo­stability, and appropriate conductivity. The preparation process and the main components of the CVD diamond micro-detector are described. The external dimensions of the packaged CVD diamond micro-detector are 15 mm × 7.8 mm × 5.8 mm. To demonstrate the performance of the detector, *K*-edge X-ray absorption fine-structure (XAFS) spectra of Cr, Fe, Cu, and Se foils were collected using the CVD diamond micro-detector and routine ion chamber. These XAFS measurements were performed at beamline 1W2B of Beijing Synchrotron Radiation Facility, covering an energy range from 5.5 to 13.5 keV. By comparison, it can be seen that the CVD diamond micro-detector shows a more excellent performance than the routine ion-chamber in recording these XAFS spectra. The successful application of the CVD diamond micro-detector in XAFS measurements shows its feasibility in recording X-ray intensity.

## Abstract 2

A polycrystalline diamond detector for XAFS measurement has been fabricated. Its volume is 2947 times smaller than a routine ion-chamber.

## 1. Introduction

The X-ray absorption fine-structure (XAFS) (Palmer *et al.*, 1996 ▸; Teo & Joy, 1981 ▸; Ankudinov *et al.*, 1998 ▸; Dalba & Fornasini, 1997 ▸; Rehr & Albers, 2000 ▸) technique can be used to probe atomic coordination structures, including electronic structure, chemical valence, local atomic structure, and chemical bond information. The acquisition of high-quality XAFS data requires the detector to have good reliability, linearity, and stability. In transmission XAFS measurements, the ionization chamber (IC) is the most popular detector, whereas, in fluorescence XAFS measurements, the solid-state detector, including the Lytle detector, is widely used. In the past decades, Si-based detectors have achieved great success and shown excellent stability. Previous studies (Storb *et al.*, 1991 ▸; Bouldin *et al.*, 1987 ▸; Dalba *et al.*, 1996 ▸) have shown that Si-based detectors can be used in synchrotron radiation beamlines with high-flux (≥10^11^ photons s^−1^) incident X-rays to record transmitted or reflected X-ray intensities. In addition to the high manufacturing cost (Dalba *et al.*, 1996 ▸; Almaviva *et al.*, 2008 ▸, 2010 ▸), the known solid-state detectors still cannot completely replace the transmission-type ICs in XAFS spectral acquisition. Single-crystal diamond is an ideal candidate for X-ray detectors because of its fast response to X-ray radiation and strong radiation resistance. Serving as a new detector, single-crystal diamond has low dark current (pA) and high signal-to-noise ratio. Especially in XAFS experiments (De Sio *et al.*, 2008 ▸) of Fe foil, a single-crystal diamond detector obtained the same high-quality data as an IC. This result confirms that diamond is suitable for X-ray detection. Up to now, the application of a diamond detector (Marinelli *et al.*, 2012 ▸) focused mainly on monitoring the beam flux and spot position. There has been almost no further application of diamond detectors in X-ray absorption spectroscopy.

With the wide applications of synchrotron radiation (SR) experimental techniques in structural research, a variety of combining techniques, including combining SR and SR techniques as well as SR and non-SR techniques, are strongly demanded by researchers. For example, XAFS/XRD (Grunwaldt & Clausen, 2002 ▸), XAFS/Raman (Briois *et al.*, 2005 ▸), XAFS/IR (Marinkovic *et al.*, 2011 ▸) and so on combining or quasi-combining techniques have been developed. In these combining techniques, ICs with a huge volume were used to collect the transmission XAFS signals. In fact, the huge volume of the IC would limit or obstruct the acquisition of experimental signals other than XAFS signals in a combining technique. When transmission XAFS spectra have to be collected, ICs with huge volume would seriously hamper the development of combining techniques that contain transmission XAFS measurements. Therefore, there is a growing desire to replace the huge-volume IC with other miniature or laminated detectors in combining techniques. The single-crystal diamond detector seems to be the best substitute for the IC. However, the intense diffraction spots produced by single-crystal diamond easily irradiate to other detectors used to record other experimental signals. Evidently, in order to prevent this interference of experimental signals, intense diffraction spots from the diamond detector should be avoided as much as possible.

In this paper, the fabrication of a diamond micro-detector will be described. In order to avoid intense diffraction spots from single-crystal diamond, chemical vapor deposition (CVD) diamond self-supporting laminae are used as the fabrication material. The CVD diamond laminae are relatively cheap, and the powder polycrystalline form can effectively overcome these intense diffraction spots. To verify the feasibility of making CVD diamond laminae into a detector, the XAFS spectra of four elemental foils (Cr, Fe, Cu, and Se) have been collected in the energy range from 5.5 to 13.5 keV. Both incident and transmission X-ray intensities will be simultaneously recorded by the polycrystalline diamond detectors. These obtained XAFS spectra will also be compared with XAFS spectra collected with a common IC for checking the performance of the polycrystalline diamond detector.

## 2. Diamond micro-detector fabrication

**Figure 1:**
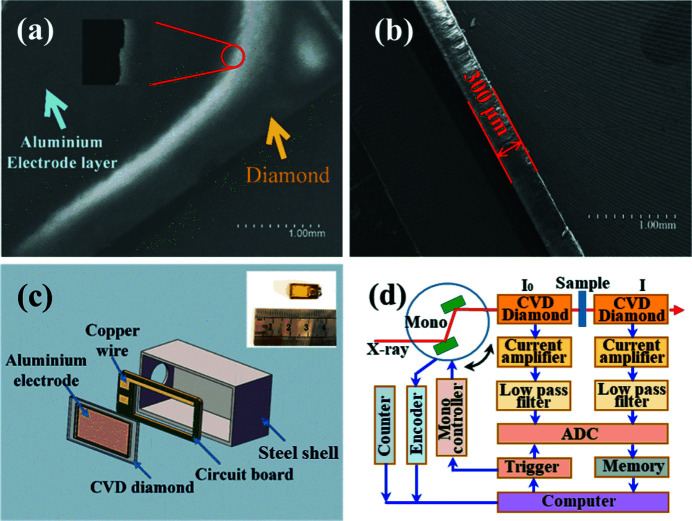
(*a*) SEM image of the full face of the CVD diamond film. (*b*) SEM image of the side face of the CVD diamond film. (*c*) Schematic map of the CVD diamond micro-detector. (*d*) Control block diagram of transmission XAFS measurements with the CVD diamond micro-detector.

First, CVD diamond films with a thickness of about 300 µm were purchased from the University of Science and Technology Beijing. Then the CVD diamond films were cut into small pieces with an area of 11 mm × 6 mm. After decarburization and degreasing of the CVD diamond pieces with H_2_SO_4_ and NH_4_OH solutions, respectively, metal aluminium was evaporated in vacuum and deposited on both sides of the diamond films to form a pair of electrodes. The areas of the aluminium electrodes are 8 mm × 5 mm and their thicknesses are less than 100 nm. To avoid the connection of the two aluminium electrodes, photoresist was applied to the four edges of the CVD diamond piece before evaporating the aluminium metal. After the deposition of the aluminium electrodes, the photoresist was washed away, resulting in exposed CVD diamond edges on the CVD diamond pieces covered by aluminium electrodes. In this way, the sizes of the aluminium electrodes were confined to a smaller range than the dimensions of the CVD diamond piece, as shown in Fig. 1 ▸(*a*). The thickness of the CVD diamond pieces is about 300 µm, as shown in Fig. 1 ▸(*b*). A hollow circuit board of size 10 mm × 5 mm × 1.5 mm was used to support the CVD diamond piece. The copper lines on the so-called circuit board were connected to the aluminium electrodes on both sides of the CVD diamond piece via gold wires, and were used to apply a bias voltage to the CVD diamond piece. The CVD diamond piece and the circuit board were then encapsulated together with a polyimide insulation film of thickness 30 µm, as shown in the illustrated photograph in Fig. 1 ▸(*c*). Finally, the packaged CVD diamond micro-detector was installed in a stainless steel shell of size 15 mm × 7.8 mm × 5.8 mm, forming a diamond micro-detector coupled with a beamstop.

As a typical application of the CVD diamond micro-detector, its control block diagram in transmission XAFS measurements is shown in Fig. 1 ▸(*d*). Similar to the routine IC, the current signal output from the CVD diamond micro-detector is firstly amplified using a weak current amplifier – Model 428 from Keithley Company. After the current (voltage) is converted to a pulse frequency signal by a V/F converter, the frequency count or the intensity is then recorded using a Model 974 counter from EG&G Company. In addition, the current or voltage signal from the CVD diamond micro-detector can also be directly recorded as the analog quantity of the corresponding X-ray intensity.

## 3. Experimental setup

The performance of the as-prepared CVD diamond micro-detector was tested at beamlines 1W2B and 4B9A of Beijing Synchrotron Radiation Facility (BSRF), where the energy range was from 5 to 18 keV. A double-crystal Si (111) monochromator was used to monochromize the incident X-ray beam with energy resolution (Δ*E*/*E*) better than 4 × 10^−4^. The photon flux was about 10^12^ photons s^−1^ at 1W2B, and the spot sizes (H × V) were 1 mm × 0.6 mm. The XAFS spectra of standard Cr, Fe, Cu, and Se foils were collected by the CVD diamond micro-detector, and were compared with those collected by a routine IC. The IC was purchased from Tianjin Jingshenfang Technology Corporation. Its overall dimensions are 110 mm × 110 mm × 352 mm (W × H × L), and its working voltage is 2000 V. With the incident X-ray energy increasing from the Cr *K*-edge (∼6 keV) to the Se *K*-edge (∼13 keV), the working gases of the IC were the mixed gases of N_2_–5%Ar, N_2_–10%Ar, and N_2_–20%Ar.

## 4. Results and discussions

**Figure 2:**
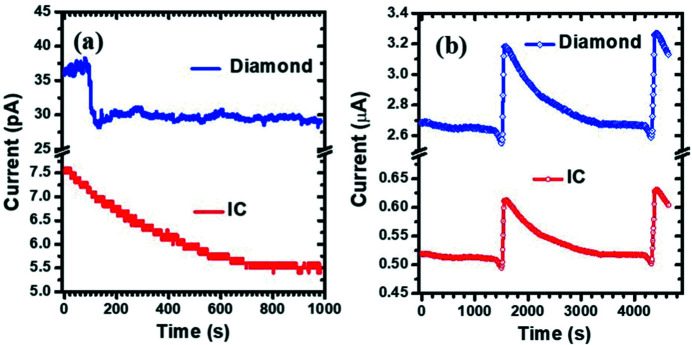
Dark-current change (*a*) and response comparison with incident X-rays (*b*) for the CVD diamond micro-detector and IC.

First, the dark currents of both IC and diamond micro-detector were monitored for 1000 s as shown in Fig. 2 ▸(*a*). The maximum dark current of the CVD diamond micro-detector was 38 pA. In the initial 80 s, its dark current slightly increased from 36 to 38 pA. Then it clearly decreased to 28 pA in the time frame from 80 to 125 s. After 125 s, the dark current was approximately stabilized at 30 pA. Except for the change of dark current in the first 125 s, the CVD diamond micro-detector can be considered to have a constant dark current of about 30 pA. However, the dark current of the IC was about one-fifth of that of the CVD diamond micro-detector. As the monitoring time increased from 0 to 1000 s, the dark current of the IC approximately decreased monotonously from 7.5 to 5.5 pA. If considering only the values of the dark currents, then the IC appears to be better than the CVD diamond micro-detector.

In order to compare further the CVD diamond micro-detector with the routine IC, their responses to incident X-rays at 8 keV were also recorded simultaneously as shown in Fig. 2 ▸(*b*). It can be seen that both curves have similar profiles. Here we have to point out that the experiments were performed in the parasitic mode of BSRF. Two abrupt changes occurred, at about 1500 s and 4400 s, corresponding to electron injection into the storage ring. Between the two injections, the response curves to incident X-ray intensity of the CVD diamond micro-detector and IC all decayed with time, resembling the decay curve of the beam intensity. In the 1590–4190 s time frame, the former attenuated monotonously from 3.2 to 2.7 µA and the latter attenuated monotonously from 0.6 to 0.5 µA. Roughly, the response of the CVD diamond micro-detector was fivefold stronger than that of the routine IC. Although the CVD diamond micro-detector has a higher dark current than the routine IC, the CVD diamond micro-detector also has a higher response to incident X-ray intensity than the routine IC. By comparing the ratios of the response current to dark current between the two kinds of detectors, it can be concluded that the CVD diamond micro-detector should have a comparable signal-to-noise ratio as the IC.

To assess further the performance of the CVD diamond micro-detector, it was also used for XAFS measurements. At beamline 1W2B of BSRF, the XAFS spectra of four elemental foils were collected in transmission mode. The four elemental foils were Cr (*E*_0_ = 5989 keV), Fe (*E*_0_ = 7112 keV), Cu (*E*_0_ = 8979 keV), and Se (*E*_0_ = 12658 keV). Their *K*-edge XAFS spectra cover an energy range from 5.5 to 13.5 keV.

**Figure 3:**
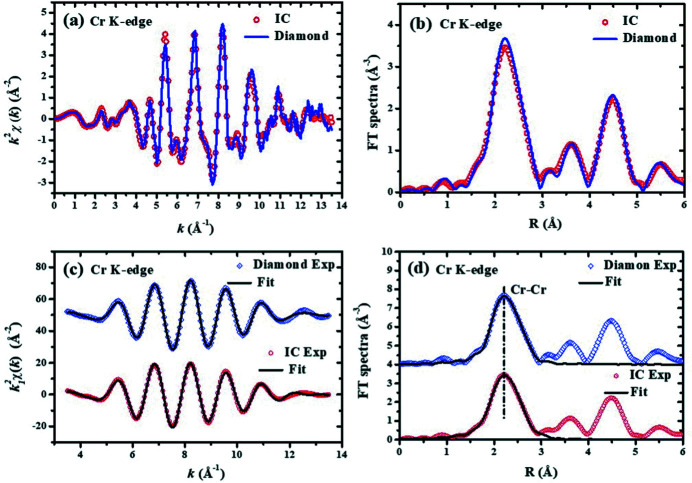
Comparison of Cr *K*-edge XAFS spectra collected with the CVD diamond micro-detector and the routine IC. (*a*) The experimental *k* ^2^χ(*k*) oscillations. (*b*) The Fourier transform (FT) spectra of *k* ^2^χ(*k*). (*c*) The isolated near-neighbor 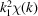 oscillations and their best-fit curves. (*d*) The FT spectra of 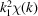 oscillations and their best-fit curves. In Figs. 3 ▸(*c*) and 3 ▸(*d*), the data of the CVD diamond micro-detector (upper) have been shifted up relative to the data of the routine IC (lower) for clarity.

X-ray absorption spectra (XAS) of the Cr *K*-edge were collected in the energy range from 5.8 to 6.8 keV. The XAFS oscillations *k* ^2^χ(*k*) were extracted as shown in Fig. 3 ▸(*a*). Their corresponding Fourier transform (FT) spectra with a *k*-range from 3.5 to 13.5 Å^−1^ are shown in Fig. 3 ▸(*b*). The XAFS oscillations 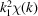 isolated from the FT spectra in the *R*-range from 1.10 to 2.98 Å are shown in Fig. 3 ▸(*c*). The near-neighbor structural parameters around the central Cr atom were obtained by fitting the XAFS data in *k*-space as shown in Fig. 3 ▸(*c*) or in *R*-space as shown in Fig. 3 ▸(*d*). In the data analysis, *Artemis* software was used to fit the experimental data by using the single-scattering extended X-ray-absorption fine-structure (EXAFS) formula (Lee *et al.*, 1981 ▸). The phase shift and backscattering amplitude of the Cr–Cr atom pair were calculated by using the *FEFF* (Newville, 2001 ▸) code. The crystallographic structure (space group 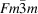 ) (Häglund *et al.*, 1993 ▸) of chromium was used as a reference, where there are eight Cr neighbors located at 2.487 Å and six Cr neighbors located at 2.883 Å. The fitting parameters of the two subshells are listed in Table 1 ▸. By comparing the experimental XAFS data collected with the CVD diamond micro-director and the routine IC, as well as their fitting curves, the signal-to-noise ratios of data collected with the two detectors are comparable; there are even no obvious differences in their fitting curves. The obtained Debye–Waller factors are also comparable. The fitting distances of the first Cr–Cr subshell are exactly the same for the two sets of XAFS data collected with the CVD diamond micro-detector and the routine IC. However, the XAFS data collected by the CVD diamond micro-detector and the routine IC show that the interatomic distance of the Cr–Cr atom pairs in the second subshell are 2.87 Å and 2.91 Å, respectively. Early research (Straumanis & Weng, 1956 ▸) showed that the distance between the central Cr and the second-neighbor Cr atom was 2.88 Å. This result reveals that the Cr–Cr distance (2.87 Å) measured by the CVD diamond micro-detector is closer to the crystallographic data (2.88 Å) than the Cr–Cr distance (2.91 Å) measured by the routine IC. Based on the ratio ( 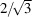 ) between the second-neighbor and the first-neighbor Cr–Cr distances in the body-centred-cubic (b.c.c.) structure of Cr metal, the Cr–Cr distance (2.87 Å) obtained with the CVD diamond micro-detector is closer to the true value than the Cr–Cr distance (2.91 Å) obtained with the routine IC. All these results imply that the XAFS data collected with the CVD diamond micro-detector seem to be more reliable and more feasible than the data collected with routine ICs.

**Figure 4:**
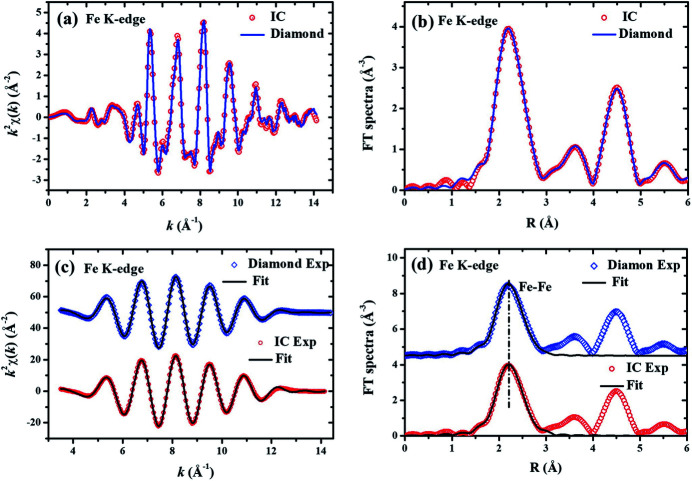
Comparison of Fe *K*-edge XAFS spectra collected with the CVD diamond micro-detector and the routine IC. (*a*) The experimental *k* ^2^χ(*k*) oscillations. (*b*) The Fourier transform (FT) spectra of *k* ^2^χ(*k*). (*c*) The isolated near-neighbor 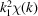 oscillations and their best-fit curves. (*d*) The FT spectra of 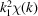 oscillations and their best-fit curves. In panels (*c*) and (*d*), the data of the CVD diamond micro-detector (upper) have been shifted up relative to the data of the routine IC (lower) for clarity.

Similarly, the Fe *K*-edge XAS of Fe foil were also recorded in the energy range from 6.9 to 7.9 keV by using the CVD diamond micro-detector and the routine IC. The XAFS oscillations *k* ^2^χ(*k*) extracted from the experimental XAS data are compared in Fig. 4 ▸(*a*), and the corresponding FT spectra of *k* ^2^χ(*k*) are compared in Fig. 4 ▸(*b*) between the CVD diamond micro-detector and the routine IC. The near-neighbor Fe–Fe coordination data 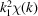 were extracted, and their fitting curves are shown in Fig. 4 ▸(*c*). The corresponding FT spectra of the 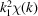 data are shown in Fig. 4 ▸(*d*). Here, the FT region was from 3.0 to 14.1 Å^−1^ and the Fourier filter region was from 1.1 to 3.1 Å. In the XAFS fitting, the backscattering amplitude and phase shift of the reference Fe–Fe atom-pairs were calculated based on the crystallographic structure (Wood *et al.*, 2008 ▸) of iron with space group 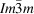 , where there were eight Fe–Fe atom-pairs with Fe–Fe distance of 2.483 Å and six Fe–Fe atom-pairs with Fe–Fe distance of 2.868 Å. The fitting parameters for the two Fe–Fe subshells are listed in Table 2 ▸. For clarity, the data collected with the CVD diamond micro-detector are shifted up in Figs. 4 ▸(*c*) and 4 ▸(*d*). From Fig. 4 ▸, it can be seen that the signal-to-noise ratios of the data collected with the CVD diamond micro-detector and the routine IC are at the same level. It cannot even be judged which fitting curve is better between the CVD diamond micro-detector and the routine IC. The first Fe–Fe subshells were all fitted to be 2.46 Å; however, the second Fe–Fe subshells were fitted to be 2.90 Å for the routine ion-chamber data or 2.84 Å for the CVD diamond micro-detector data. Similarly, the b.c.c. structure of Fe metal determines that the ratio between the second-neighbor and the first-neighbor Fe–Fe distances should be 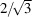 . Evidently, the XAFS data collected with the CVD diamond micro-detector seem to be more reliable and more accurate.

**Figure 5:**
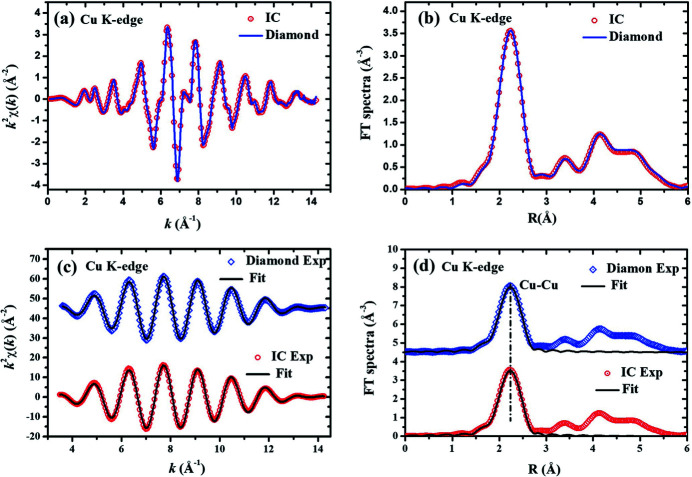
Comparison of Cu *K*-edge XAFS spectra collected with the CVD diamond micro-detector and the routine IC. (*a*) The experimental *k* ^2^χ(*k*) oscillations. (*b*) The Fourier transform (FT) spectra of *k* ^2^χ(*k*). (*c*) The isolated near-neighbor 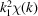 oscillations and their best-fit curves. (*d*) The FT spectra of 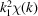 oscillations and their best-fit curves. In panels (*c*) and (*d*), the data of the CVD diamond micro-detector (upper) have been shifted up relative to the data of the routine IC (lower) for clarity.

Furthermore, the Cu *K*-edge XAFS data from 8.7 to 9.8 keV and the Se *K*-edge XAFS data from 12.45 to 13.45 keV were also collected with the CVD diamond micro-detector and the routine IC. The Cu *K*-edge and the Se *K*-edge XAFS data are shown in Figs. 5 ▸ and 6 ▸, respectively. The Cu *K*-edge (or the Se *K*-edge) XAFS spectra *k* ^2^χ(*k*) in the *k*-range from 3.5 to 14.1 Å^−1^ were Fourier transformed into *R*-space, and then the near-neighbor coordination peak in the *R*-range from 1.20 to 2.89 Å (or from 1.20 to 2.51 Å) were Fourier isolated into *k*-space again to obtain the near-neighbor Cu–Cu (or Se–Se) XAFS curves. Different from the Cr and Fe foils, the first near-neighbor coordination peaks in the FT spectra of the Cu *K*-edge (or the Se *K*-edge) XAFS data contain only one Cu–Cu (or Se–Se) coordination shell. The reference crystallographic structure was space group 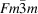 (Smura *et al.*, 2011 ▸) for Cu foil or space group *P*3_1_21 (Boeré & Hill, 2017 ▸) for Se foil. That is to say, there are twelve Cu–Cu atom-pairs with a Cu–Cu distance of 2.556 Å or there are three Se–Se atom-pairs with an Se–Se distance of 2.380 Å. The fitting parameters of the first near-neighbor coordination peaks for the Cu and Se foils are listed in Table 3 ▸. By comparing, it can be found that the fitting parameters are all in excellent agreement with the crystallographic data for both Cu and Se foils. These results demonstrate again that the CVD diamond micro-detector is as suitable for XAFS measurements as the routine IC.

In this study, *K*-edge X-ray absorption spectra of Cr, Fe, Cu and Se foils were collected, covering an X-ray energy range from 5.5 to 13.5 keV. These XAFS spectra collected with the CVD diamond micro-detector have almost the same signal-to-noise ratio as those collected with the routine IC. The XAFS fitting parameters for the Cr, Fe Cu and Se foils are in excellent agreement with the crystallographic structures. At least, the near-neighbor bond lengths obtained from the data of the CVD diamond micro-detector seem to be more reliable than those obtained from the data of the routine IC. Evidently, the CVD diamond micro-detector can replace the routine IC for the *K*-edge XAFS measurements of transition elements. By changing the thickness of the diamond film, the CVD diamond micro-detector could also be appropriate for X-ray intensity measurements in other energy ranges. It should be noted that the CVD diamond micro-detector has smaller dimensions – it is especially thinner along the X-ray path – than the routine IC. Therefore, the CVD diamond micro-detector will be more suitable for some SR combining techniques.

## 5. Conclusion

A micro-detector composed of CVD diamond film has been successfully fabricated. Such a CVD diamond micro-detector can replace the routine IC and may obtain almost the same signal-to-noise ratio as the routine IC in XAFS measurements. This CVD diamond micro-detector has been identified to be effective in the energy range from 5.5 to 13.5 keV for X-ray intensity collection. In particular, it has obvious advantages in the application of SR combined techniques owing to its small dimensions.

## Figures and Tables

**Figure 6:**
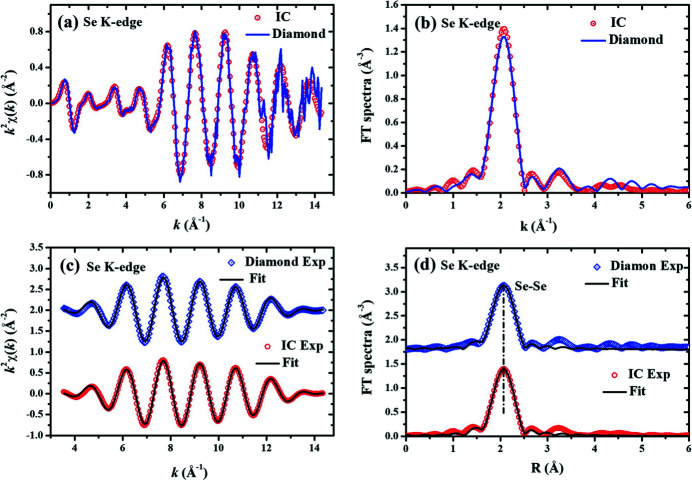
Comparison of Se *K*-edge XAFS spectra collected with the CVD diamond micro-detector and the routine IC. (*a*) The experimental *k* ^2^χ(*k*) oscillations. (*b*) The Fourier transform (FT) spectra of *k* ^2^χ(*k*). (*c*) The isolated near-neighbor 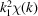 oscillations and their best-fit curves. (*d*) The FT spectra of 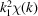 oscillations and their best-fit curves. In panels (*c*) and (*d*), the data of the CVD diamond micro-detector (upper) have been shifted up relative to the data of the routine IC (lower) for clarity.

**Table 1:** Best-fit parameters of the near-neighbor structure around Cr in Cr foil The fitting results are compared for the XAFS data collected with the CVD diamond micro-detector and the routine IC.

Parameters	Subshell-1: Cr–Cr, *N* = 8		Subshell-2: Cr–Cr, *N* = 6	
	IC	Diamond	IC	Diamond
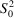	0.67	0.60	0.85	0.75
*R* (Å)	2.47	2.47	2.91	2.87
σ^2^ × 10^3^ (Å^2^)	5.41	3.48	4.42	3.49
Δ*E* (eV)	1.64	2.95	5.92	4.74

**Table 2:** Best-fit parameters of the near-neighbor structure around Fe in Fe foil The fitting results are compared for the XAFS data collected with the CVD diamond micro-detector and the routine IC.

Parameters	Subshell-1: Fe–Fe, *N* = 8		Subshell-2: Fe–Fe, *N* = 6	
	IC	Diamond	IC	Diamond
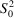	0.77	0.85	0.78	0.89
*R* (Å)	2.46	2.46	2.90	2.84
σ^2^ × 10^3^ (Å^2^)	5.93	5.81	4.48	7.20
Δ*E* (eV)	4.94	6.37	8.36	5.99

**Table 3:** Best-fit parameters of the near-neighbor structure around Cu in Cu foil and around Se in Se foil The fitting results are compared for the XAFS data collected with the CVD diamond micro-detector and the routine IC.

Parameters	Subshell-1: Cu–Cu, *N* = 12		Subshell-1: Se–Se, *N* = 3	
	IC	Diamond	IC	Diamond
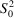	0.79	0.75	0.68	0.77
*R* (Å)	2.53	2.53	2.37	2.38
σ^2^ × 10^3^ (Å^2^)	7.72	7.33	4.21	5.15
Δ*E* (eV)	4.00	4.29	7.71	7.90

## References

[bb2] Almaviva, S., Marinelli, M., Milani, E., Prestopino, G., Tucciarone, A., Verona, C., Verona-Rinati, G., Angelone, M., Lattanzi, D., Pillon, M., Montereali, R. M. & Vincenti, M. A. (2008). *J. Appl. Phys.* **103**, 054501.

[bb1] Almaviva, S., Marinelli, M., Milani, E., Prestopino, G., Tucciarone, A., Verona, C., Verona-Rinati, G., Angelone, M., Pillon, M., Dolbnya, I., Sawhney, K. & Tartoni, N. (2010). *J. Appl. Phys.* **107**, 014511.

[bb3] Ankudinov, A. L., Ravel, B., Rehr, J. J. & Conradson, S. D. (1998). *Phys. Rev. B*, **58**, 7565–7576.

[bb4] Boeré, R. T. & Hill, N. D. D. (2017). *CrystEngComm*, **19**, 3698–3707.

[bb5] Bouldin, C. E., Forman, R. A. & Bell, M. I. (1987). *Rev. Sci. Instrum.* **58**, 1891–1894.

[bb6] Briois, V., Lützenkirchen-Hecht, D., Villain, F., Fonda, E., Belin, S., Griesebock, B. & Frahm, R. (2005). *J. Phys. Chem. A*, **109**, 320–329. 10.1021/jp046691t 16833350

[bb7] Dalba, G. & Fornasini, P. (1997). *J. Synchrotron Rad.* **4**, 243–255. 10.1107/S0909049597006900 16699237

[bb8] Dalba, G., Fornasini, P., Soldo, Y. & Rocca, F. (1996). *J. Synchrotron Rad.* **3**, 213–219. 10.1107/S0909049596006073 16702681

[bb9] De Sio, A., Bocci, A., Pace, E., Castellano, C., Cinque, G., Tartoni, N. & D’Acapito, F. (2008). *Appl. Phys. Lett.* **93**, 083503.

[bb10] Grunwaldt, J. D. & Clausen, B. S. (2002). *Top. Catal.* **18**, 37–43.

[bb11] Häglund, J., Fernández Guillermet, F., Grimvall, G. & Körling, M. (1993). *Phys. Rev. B*, **48**, 11685–11691. 10.1103/physrevb.48.11685 10007503

[bb12] Lee, P. A., Citrin, P. H., Eisenberger, P. & Kincaid, B. M. (1981). *Rev. Mod. Phys.* **53**, 769–806.

[bb13] Marinelli, M., Milani, E., Prestopino, G., Verona, C., Verona-Rinati, G., Angelone, M., Pillon, M., Kachkanov, V., Tartoni, N., Benetti, M., Cannatà, D. & Di Pietrantonio, F. (2012). *J. Synchrotron Rad.* **19**, 1015–1020. 10.1107/S0909049512038186 23093764

[bb14] Marinkovic, N. S., Wang, Q. & Frenkel, A. I. (2011). *J. Synchrotron Rad.* **18**, 447–455. 10.1107/S0909049511005802 21525654

[bb15] Newville, M. (2001). *J. Synchrotron Rad.* **8**, 322–324. 10.1107/s0909049500016964 11512767

[bb16] Palmer, B. J., Pfund, D. M. & Fulton, J. L. (1996). *J. Phys. Chem.* **100**, 13393–13398.

[bb17] Rehr, J. J. & Albers, R. C. (2000). *Rev. Mod. Phys.* **72**, 621–654.

[bb18] Smura, C. F., Parker, D. R., Zbiri, M., Johnson, M. R., Gál, Z. A. & Clarke, S. J. (2011). *J. Am. Chem. Soc.* **133**, 2691–2705. 10.1021/ja109553u 21302927

[bb19] Storb, C., Dedek, U., Weber, W., Lengeler, B. & Schuster, M. (1991). *Nucl. Instrum. Methods Phys. Res. A*, **306**, 544–548.

[bb20] Straumanis, M. E. & Weng, C. C. (1956). *Am. Miner.* **41**, 437–448.

[bb21] Teo, B. K. & Joy, D. C. (1981). *EXAFS Spectroscopy – Techniques and Applications.* Springer.

[bb22] Wood, I. G., Vočadlo, L., Dobson, D. P., Price, G. D., Fortes, A. D., Cooper, F. J., Neale, J. W., Walker, A. M., Marshall, W. G., Tucker, M. G., Francis, D. J., Stone, H. J. & McCammon, C. A. (2008). *J. Appl. Cryst.* **41**, 886–896.

